# Effect of balanced protein energy supplementation during pregnancy on birth outcomes

**DOI:** 10.1186/1471-2458-11-S3-S17

**Published:** 2011-04-13

**Authors:** Aamer Imdad, Zulfiqar A Bhutta

**Affiliations:** 1 Division of Women & Child Health, The Aga Khan University, Karachi, Pakistan

## Abstract

**Background:**

The nutritional status of the mother prior to and during pregnancy plays a vital role in fetal growth and development, and maternal undernourishment may lead to adverse perinatal outcomes including intrauterine growth restriction (IUGR). Several macronutrient interventions had been proposed for adequate protein and energy supplementation during pregnancy. The objective of this paper was to review the effect of balanced protein energy supplementation during pregnancy on birth outcomes. This paper is a part of a series of reviews undertaken for getting estimates of effectiveness of an intervention for input to Lives Saved Tool (LiST) model.

**Methods:**

A literature search was conducted on PubMed, Cochrane Library and WHO regional data bases to identify randomized trials (RCTs) and quasi RCTs that evaluated the impact of balanced protein energy supplementation in pregnancy. Balanced protein energy supplementation was defined as nutritional supplementation during pregnancy in which proteins provided less than 25% of the total energy content. Those studies were excluded in which the main intervention was dietary advice to pregnant women for increase in protein energy intake, high protein supplementation (i.e. supplementation in which protein provides at least 25% of total energy content), isocaloric protein supplementation (where protein replaces an equal quantity of non-protein energy content), or low energy diet to pregnant women who are either overweight or who exhibit high weight gain earlier in gestation. The primary outcomes were incidence of small for gestational age (SGA) birth, mean birth weight and neonatal mortality. Quality of evidence was evaluated according to the Child Health Epidemiology Reference group (CHERG) adaptation of Grading of Recommendations Assessment, Development and Evaluation **(**GRADE) criteria.

**Results:**

The final number of studies included in our review was eleven comprising of both RCTs and quasi-RCTs. Our meta-analysis indicates that providing pregnant females with balanced protein energy supplementation resulted in a significant reduction of 31 % in the risk of giving birth to small for gestational age infants (Relative risk (RR) =0.69, 95% Confidence interval (CI) 0.56 to 0.85). This estimate had been recommended for LiST as a proxy for reduction in IUGR. Pooled results for mean birth weight showed that balanced protein supplemented group gained more weight compared to control [Mean difference 59.89 g, 95 % CI 33.09-86.68]. This effect was more pronounced in malnourished women compared to adequately nourished women. There was no statistically significant effect of balanced protein energy supplementation on neonatal mortality (RR= 0.63, 95% CI 0.37 to 1.06).

**Conclusion:**

Providing pregnant females with balanced protein energy supplementation leads to reduction in risk of small for gestational age infants, especially among undernourished pregnant women. Given these findings, we can recommend balanced protein energy supplementation as an intervention among undernourished women for inclusion in the LiST model with a point estimate of 31% [95% CI 15% to 44%] reduction in IUGR.

## Introduction

According to an estimate, approximately 30 million newborns per year are affected with intrauterine growth restriction (IUGR) in developing countries [1]. This rate is six times higher than in developed countries. The highest burden of prevalence of term low birth weight/IUGR lies in Asia (75%), mainly South East Asia, followed by Africa (20%) and Latin America (5%) [1]. IUGR is associated with an increase in perinatal mortality and morbidities such hypothermia, hypoglycemia, prematurity etc [2]. Babies with restricted intrauterine growth are more likely to have poor cognitive development during childhood leading to neurologic impairment in adulthood and also an increased risk of cardiovascular, pulmonary and renal diseases later in life [3,4]. It has now been shown that poor maternal nutritional status at conception and inadequate maternal nutrition during pregnancy can result in IUGR [5,6]. The main focus of maternal and fetal nutrition during pregnancy is to achieve appropriate energy intakes (in the form macronutrients) and ensuring that the intakes of specific nutrients (like vitamin and minerals) are adequate to meet maternal and fetal needs [7].

Several macro/micronutrient nutritional interventions have been proposed and evaluated in accordance with the maternal needs during pregnancy [8]. Some of the macronutrient interventions include dietary advice to pregnant women, balanced protein energy supplementation, high protein, isocaloric protein supplementation, prescribing low energy diet to pregnant women who are either overweight or who exhibit high weight gain earlier in gestation [9-11]. Among these interventions, balanced protein energy supplementation is considered as one of the most promising macronutrient interventions for prevention of adverse perinatal outcomes including IUGR [9].

Previous reviews on maternal nutritional supplementation during pregnancy have shown that balanced protein energy supplementation has a positive impact on both maternal and perinatal birth outcomes [9,12]. These reviews concluded that balanced protein energy supplementation leads to a modest increase in maternal weight gain during pregnancy and birth weight of the baby. It was also associated with a significant reduction in small-for-gestational-age (SGA) infants and stillbirths and with a non-significant reduction in neonatal mortality.

The purpose of this review was to evaluate the effectiveness of balanced protein energy supplementation during pregnancy in reducing IUGR and to get a point estimate for its inclusion in the Lives Saved Tool (LiST). This is achieved through qualitative assessment of the available evidence by Grading of Recommendations Assessment, Development and Evaluation **(**GRADE) criteria [13] and quantitative inferences based on rules developed by the Child Health Epidemiology Reference Group (CHERG) to collective mortality and morbidity outcomes [14]. For more details of the review methods, the adapted GRADE approach or the LiST model, see the methods paper [14].

## Methods

### Searching

To assess the evidence of impact of maternal balanced protein energy supplementation on pregnancy outcomes, a literature search was conducted on PubMed, the Cochrane library, and the World Health Organization Regional Databases. The last date of search was February 28, 2010. The following search strategy was applied for the search of articles on PubMed: (Pregnancy* OR maternal OR "Mothers"[Mesh] OR "Pregnancy"[Mesh] OR "Pregnant Women"[Mesh]) AND (balanced OR protein OR energy) AND (supplement*). This search strategy was modified accordingly for the searches on other databases as some of the data bases don’t take mesh terms used on PubMed. The bibliographies of available reviews and meta-analyses were also hand searched to look for any additional studies.

### Selection (inclusion/exclusion criteria)

All randomized and quasi-randomized controlled trials assessing impact of balanced protein energy supplementation on pregnancy outcomes were eligible for inclusion, irrespective of language, geographical region or publication status. Balanced protein energy supplementation was defined as nutritional supplementation during pregnancy in which proteins provided less than 25% of the total energy content [12]. Those studies were excluded in which the main intervention was dietary advice to pregnant women for increase in protein energy intake, high protein supplementation (i.e. supplementation in which protein provides at least 25% of total energy content), isocaloric protein supplementation (where protein replaces an equal quantity of non-protein energy content), or low energy diet to pregnant women who are either overweight or who exhibit high weight gain earlier in gestation. Small for gestational age was defined as a baby whose weight was below the 10^th^ percentile for its gestational age [15], while neonatal mortality was defined as death of a live born infant within the first 28 days of life [16].

### Abstraction, analyses, and summary measures

Data from all the included studies were double abstracted onto a standardized form for each outcome of interest. The primary outcomes of interest were small for gestational age babies, mean birth weight and neonatal mortality. We abstracted key variables with regards to the study identifiers and context (i.e. study population, type and duration of supplementation etc), study design (i.e. sequence generation, allocation concealment, blinding and attrition), sample size and data on primary outcomes. For dichotomous outcomes, the total number of participants for each group and the number of participants experiencing an event was extracted. For continuous data, means with their standard deviations were abstracted. Each included study was assessed and graded according to the CHERG adaptation of the GRADE criteria [13,14]. In this method of qualitative evaluation, a randomized or a cluster randomized trial was given a high score initially and the grade was subsequently decreased or increased depending on strengths or limitations of study. Each study was assigned a quality grade of “high” “moderate” “low” or “very low” and studies getting a score of “very low” quality were excluded from the analysis.

For outcomes, where data were available from more than one study, we conducted meta-analyses and reported pooled relative risk (RR) and corresponding 95% confidence interval (CI). Assessment of statistical heterogeneity among the pooled trials was done by visual inspection of forest plots, by the Chi square (p-value) and by calculating the I^2^ statistic (calculated as I^2^ =100% x (Q-df )/Q; where Q is Cochrane’s heterogeneity statistic and df is the degrees of freedom). Heterogeneity was taken as substantial if p-value of Chi square was < 0.10, I^2^ exceeded 50% and visual inspection of forest plots was indicative. Reasons for heterogeneity were explored by doing a sensitivity analysis by taking out studies of moderate or low quality. Fixed models were used for primary analysis. In case of cluster randomized controlled trials, it was taken into account whether the study subjects were randomized in groups (i.e. clusters) or at individual level. Preference was given to cluster adjusted values given in the study and if results were not adjusted for cluster randomization, sample size were adjusted by using an estimate of the intra-cluster correlation co-efficient (ICC) derived from the trial (if possible), or were inferred from similar studies [17]. Pooled estimates of the evaluated outcome measures were calculated by the generic inverse variance method. This method is a common and simple version of the meta-analysis procedure and is so named because the weight given to each study is chosen to be the inverse of the variance of the effect estimate (i.e. one over the square of its standard error) [17]. All analyses were conducted using software Review Manager version 5 [18].

Recommendations for Lives Saved Tool (LiST) were based on qualitative grading of the overall evidence according to the GRADE criteria and quantitative attributes according to the CHERG guidelines [14]. The quality grade of *overall* evidence from all the included studies for each outcome, was assessed on the basis of volume and consistency of the overall evidence, the size of the pooled effect and the strength of the statistical evidence for an association between the intervention and outcome [14].

## Results

### Trial flow

**Figure 1:**
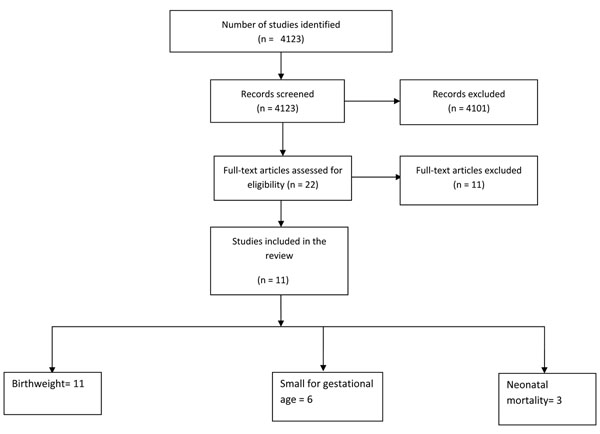
Flow diagram showing identification of studies evaluating effect of balanced protein energy supplementation during pregnanancy

We identified 4123 titles from searches conducted in all databases (Figure 1). After screening the titles and abstracts, 22 studies were identified that addressed protein energy supplementation during pregnancy. Six of these studies were excluded because the only intervention in these studies was dietary advice about increase in protein energy content [19-24]. Two studies were excluded because they addressed high or iso-caloric protein energy supplement [25,26]. Fourteen studies addressed balanced protein energy supplementation during pregnancy [27-40]. Two of these studies were excluded because both the groups received food supplementation (high versus low energy) [33,34]. Another study was excluded because of ‘very low’ quality [40]. Thus a total of eleven studies were included in this review [27-32,35-39].

### Study characteristics

Additional File 1 presents the characteristics of included studies. Five of the included studies were from developing countries [28,30,32,36,41] and six were from developed countries [27,29,31,37-39]. In seven of the included studies, women were undernourished and were at risk of having a low birth weight baby [27-30,32,35,37]. However, the method of assessment of maternal nutrition status and risk of low birth weight was very variable in the included studies. Additional File 2 presents the risk of bias table. Some of the studies were at increased risk of bias for sequence generation and allocation concealment and the grades were adjusted accordingly.

### Quantitative data synthesis

**Figure 2:**
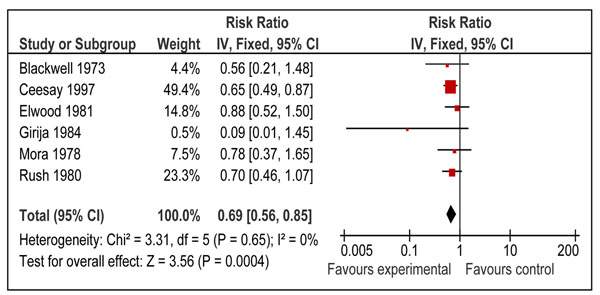
Effect of balanced protein energy supplementation during pregnancy on risk of small-for-gestational age births

Table 1 reports the overall quality grading of the outcomes and results of the corresponding meta-analyses for outcomes of interest for inclusion in the LiST. Data on small for gestational age was available from six studies [28,30-32,37,41] and the pooled results from these studies (Figure 2) indicated that this intervention was associated with an overall significant reduction in the risk of small-for-gestational age babies (RR = 0.69, 95% CI: 0.56 - 0.85). There was no heterogeneity in the pooled estimate and all the studies were showing a trend towards reduction. The overall quality grade of this outcome was that of “moderate” level. On the basis of volume, consistency and statistical significance, this estimate has been recommended as a proxy for reduction in IUGR for the LiST model. More details about these recommendations are presented in the discussion section.

**Table 1:** Results of pooled analysis and qualitative grading according to GRADE criteria for outcomes of interest for inclusion in the LiST:

Quality Assessment						Summary of Findings		
				Directness		No of patients		Effect
				
No of studies	Design	Limitations	Consistency	Generalizability to Population of Interest	Generalizability to Intervention of interest	Intervention	Control	Relative Risk (95% CI)

**Impact of balance protein energy supplementation on small for gestational age: Quality of evidence - Moderate**								

**6**[28,30-32,35,37]	RCTs / Cluster RCT/ Quasi RCTs	Two studies were quasi experimental trial. Sequence generation and allocation concealment was not adequate in some of the included studies.	No heterogeneity in the pooled data (I^2^ =0%). p= 0.65	Studies conducted in both developed and developing countries.	Protein content of Supplement for intervention group ranged from 30 g to 44 g per day. The protein content provided < 25 % of total energy content.	142	193	0.69 (0.56-0.85)

**Impact of balance protein energy supplementation on neonatal mortality: Quality of evidence - Low**								

**3**[30,37,41]	RCTs/ Cluster RCT/ Quasi RCTs	One quasi-experimental design. Allocation concealment was not adequate for one of the included cluster randomized controlled trial. Large loss to follow up in included studies.	No heterogeneity. (I^2^ =0) p=0.81	One study from developed country and two from developing countries	Protein content of Supplement for intervention group ranged from 30 g to 44 g per day. The protein content provided < 25 % of total energy content.	23	33	0.63 (0.37-1.06)

**Figure 3:**
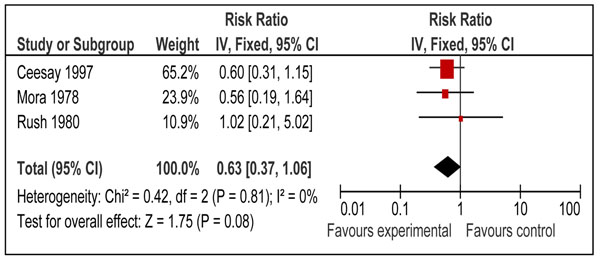
Effect of balanced protein energy supplementation during pregnancy on risk of neonatal mortality

Three studies also reported the impact of balanced protein energy supplementation during pregnancy on neonatal mortality [30,37,41]. The risk of neonatal mortality was lower with balanced protein energy supplementation during pregnancy (RR = 0.63, 95% CI: 0.37 to 1.06); however the results did not reach statistical significance (Figure 3).

**Figure 4:**
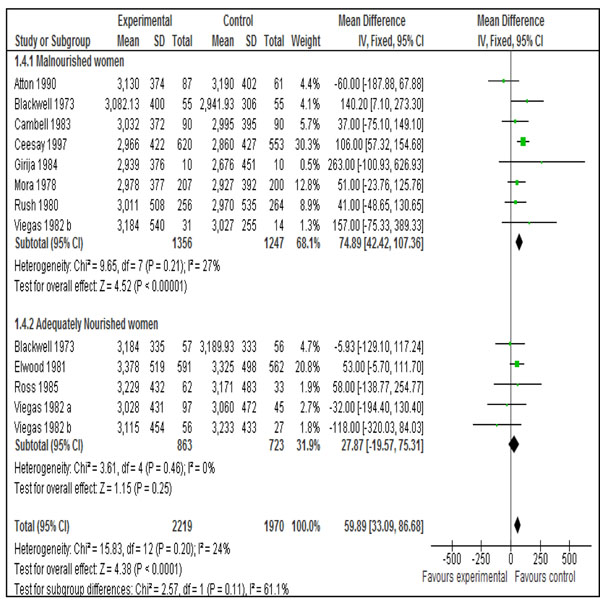
Effect of balanced protein energy supplementation during pregnancy on birthweight:

Data on mean birth weight was available from all the eleven included studies [27-32,35-39]. Pooled results showed that balanced protein supplemented group gained more weight compared to control [Mean difference 59.89 g, 95 % CI 33.09-86.68]. This effect was more pronounced in malnourished women compared to adequately nourished women (Figure 4).

## Discussion

Several reviews have concluded that the adverse birth outcome could be directly related to poor maternal nutritional status [9,12,42,43]. The maternal malnutrition during pregnancy is commonly attributed to inadequate dietary intake during pregnancy or undernutrition at the time of conception [8,33,44,45]**.** Intrauterine growth restriction represents pathological inhibition of fetal growth and failure of the fetus to attain its growth potential [46]. IUGR has also been used as a marker to assess complications of pregnancy with considerable impact on long term outcomes [3]. There is however, no standard definition of IUGR. It has been defined as a birth weight < 2 standard deviations below the median for gestational age, whereas others use a threshold of 3rd or 5th percentile of weight for age for the given population [3,47]. The term small for gestational age (SGA), usually defined as having a birth weight below the 10th percentile of an accepted reference standard, is often used as a proxy measure for IUGR [47]. These two terms are however not synonymous as some SGA infants may merely represent the lower tail of the 'normal' fetal growth distribution, while others who have been affected *in utero* by an inadequate nutritional milieu or other growth-inhibiting influences may nevertheless have a birth weight that is 'appropriate' for gestational age (AGA) [47]. Even though the terms SGA and IUGR are not synonymous, there is correlation between the two and the higher the SGA rate, the greater the likelihood that SGA is a result of IUGR [15]. Consonant with the cohort model approach first employed in the Lancet series on maternal and child undernutrition [48], the LiST tool employs a similar approach and uses the effect of various maternal interventions on SGA which is considered as a proxy measure for IUGR and an indirect cause of mortality and morbidity in children [14].

Our analysis indicates that there is a 31% [95% CI 15% to 44%] reduction in the risk of delivering a SGA infant when mothers were provided with balanced protein energy supplementation during pregnancy. We recommend this point estimate for reduction in the risk of SGA births for use in the LiST model as effectiveness of balanced protein energy supplementation in reducing IUGR. All the included the studies were found to be consistent and demonstrated little heterogeneity on meta-analysis (Figure 2). Participants in all the included studies for this analysis were undernourished except in the study by Elwood et al [31]. If we exclude this study, relative risk becomes 0.66 (95 % CI 0.53-0.82) which is not very different from the primary pooled estimate. This means that results for reduction in risk of SGA do not change significantly by excluding this study and can be generalized to undernourished women. Our results are also comparable with that of previous reviews assessing nutrition interventions during pregnancy [8,9,12]. The overall quality of evidence for this outcome was of a ‘moderate’ level due to the quasi randomized design of some included studies and recommended estimates being based on studies from both developing and developed countries. A score of “moderate” means that the reviewers are confident of the inclusion of the intervention in the model and given available information are presenting the best estimate of effectiveness. Additional research may alter the size of the effect but is not likely to change the inclusion in the model [14]. The direction and magnitude of effect size for neonatal mortality was similar to that of IUGR however the boundaries of confidence interval included unity.

There was diversity in the type food used for delivery of protein and energy among studies and included chocolate colored liquid supplements, biscuits, milk, sesame cakes, enriched bread and beverages etc. The control group was either simply observed with no intervention or given mineral and vitamin supplements only. These variations in the supplement used are understandable keeping in mind diversity of study sites and traditional foods used during pregnancy in the particular study area.

Effect of balanced protein energy supplementation seemed more pronounced in malnourished women. Our pooled results for mean change in birth weight showed that malnourished women benefited the most from balanced protein energy supplementation [mean difference 74.89 g, 95 % CI 42.42-107.36] and there was no statistically significant effect in adequately nourished women (mean difference=27.87g, 95% CI= - 19.57, 75.31). This is however contrary to pooled results by Kramer and Kakuma [12] who showed that there is no overall beneficial effect of balanced protein energy supplementation on birth weight. This was because they included the study by Kardjati et al. [33] and we excluded it. We excluded this study because both the groups were supplemented with food (high vs. low energy) and it was difficult to separate the effect of supplementary food in the control group to establish an association between intervention and the outcomes.

Our review has certain limitations. Given that the studies were conducted in both developed and developing countries, it is difficult to generalize the effect of balanced protein energy supplementation to developing countries. However, an important thing to note is that four [27,29,37,38] out of six studies from developed countries included women who were undernourished as were that of developing countries. It means that population under study in most of the studies was similar i.e. undernourished. However, this finding should be interpreted with caution as standardized maternal body mass index cut offs were not used in these studies. The 11 included trials were of variable methodological quality; the major methodological defects being the failure to provide details of allocation concealment, addressing the missing data etc. These may have biased the results in favor of intervention.

In summary, given the beneficial effects of balanced protein energy supplementation in reducing intrauterine growth restriction, and taking into account the long term sequel of IUGR, it is desirable that this intervention should be scaled up in developing countries. Given widespread maternal undernutrition in the developing world and the associated risk of being born SGA/IUGR [49], we believe that balanced energy protein supplementation can be appropriately recommended as an intervention among malnourished pregnant women and food insecure populations [1,3,9]**.**

## Competing interests

The authors declare that they have no competing interests.

## Authors' contributions

Professor Zulfiqar Ahmed Bhutta gave the idea of the review and secured support. Dr Aamer Imdad did the literature search, data extraction and wrote the manuscript along with Professor Bhutta.

## Supplementary Material

Additional File 1 Characteristics of included studies Click here for file

Additional File 2 Risk of bias among studies included studies in this analysis Click here for file
